# Leisure Time Physical Activity Reduces the Risk for Stroke in Adults: A Reanalysis of a Meta-Analysis Using the Inverse-Heterogeneity Model

**DOI:** 10.1155/2019/8264502

**Published:** 2019-06-02

**Authors:** George A. Kelley, Kristi S. Kelley

**Affiliations:** Department of Biostatistics, West Virginia University, Morgantown, WV, USA

## Abstract

**Objective:**

Apply more robust and additional analyses to a previous meta-analysis that reported statistically significant associations between leisure time physical activity (LTPA) and stroke.

**Methods:**

A reanalysis of a previous meta-analysis that included nine prospective cohort studies representing 269,594 men and women 25-84 years of age and in which the association between LTPA and incident stroke was examined. Follow-up periods ranged from 7.7 to 32.0 years. Relative risks (RR) from each study were pooled using the inverse-heterogeneity model. Heterogeneity was examined using the Q statistic, inconsistency using* I*^2^, and small-study effects using Doi plots and the LFK index. Influence and cumulative meta-analysis were also conducted.

**Results:**

Using low LTPA as the reference, moderate LTPA was associated with a statistically significant reduction in the risk for stroke in men (RR = 0.79, 95% CI = 0.65 to 0.95) and a trend in women (RR = 0.88, 95% CI = 0.78 to 1.0). High LTPA was associated with a statistically significant reduction in the risk for stroke in both men (RR = 0.72, 95% CI = 0.60 to 0.86) and women (RR = 0.78, 95% CI = 0.66 to 0.92). No statistically significant heterogeneity was observed and inconsistency was low. However, potential small-study effects were observed. With each study deleted once, results remained statistically significant. Cumulative meta-analysis demonstrated stability in results since at least 2005.

**Conclusions:**

Leisure time physical activity is associated with a reduced risk of stroke in both men and women. However, the small-study effects observed suggest the possibility that results may be exaggerated.

## 1. Introduction

Cerebrovascular disease is a major public health problem worldwide. In 2013, stroke (hemorrhagic and ischemic) was the second most common cause of all deaths globally (11.8%), after ischemic heart disease (14.8%) [1, 2]. In addition, when compared to the year 1990, the overall stroke burden with respect to the absolute number of people affected has also increased worldwide [1, 2]. Based on ICD-10 codes 160-169, cerebrovascular disease in the United States (US) was the fifth leading cause of mortality in 2015, with an estimated 140,323 deaths (5.2% of all deaths) [3]. In addition, an estimated 7.2 million US adults 20 years of age and older reported having a stroke in 2014, with overall prevalence estimated at 2.7% [4]. Furthermore, approximately 795,000 people (46.5% males, 53.5% females) experienced a new or recurrent stroke in 2015, the majority of which were ischemic [4]. Not surprisingly, the costs of stroke are also high. For example, for the period 2013 to 2014, the annual direct and indirect costs of stroke in the US were estimated at $40.1 billion, with an expected doubling from $36.7 billion to $94.3 billion between 2015 and 2030 [4]. In 27 European countries, the annual economic cost of stroke was estimated at $30.7 billion [5].

One of the major nonpharmacologic approaches for the prevention of stroke is physical activity, especially leisure time physical activity (LTPA) [4]. To support this contention, a previous systematic review with meta-analysis that included an examination of the association between leisure time physical activity (LTPA) and incident fatal and nonfatal stroke (ischemic or hemorrhagic) reported, based on a random-effects model, a statistically significant reduction in the relative risk (RR) of stroke as a result of moderate and high LTPA in men (moderate = 27%; high = 29%) and high LTPA in women (22%) [6]. A trend for a statistically significant reduction was observed for moderate LTPA in women (11%). For all analyses, no statistically significant small-study effects (publication bias, etc.) were found for any of the analyses based on Begg's test for asymmetry [7]. However, since the time of this publication [6], a more robust model, the inverse-heterogeneity (IVhet) model has been developed for pooling the results of an aggregate data meta-analysis [8]. In addition, alternative recommendations are now available for assessing small-study effects [9], as well as a very recent qualitative and quantitative approach that is considered more robust for assessing small-study effects [10]. Furthermore, this previous meta-analysis (1) did not report between-study heterogeneity and inconsistency results, an important consideration when trying to reach conclusions based on the results of a meta-analysis [6], (2) did not conduct influence analysis with each study deleted from the model once to see if any one study had a significant effect on the direction of findings, an important factor given the limited number of effect sizes reported (N = 13), and (3) did not conduct cumulative meta-analysis, ranked by year, to examine cumulative findings over time [11]. Thus, given the need to provide more robust estimates regarding the potential benefit of LTPA on incident stroke, a major public health problem, the purpose of this short study was to use data from this previous meta-analysis [6] and apply more robust as well as additional analyses on the association between LTPA and stroke incidence.

## 2. Materials and Methods

### 2.1. Data Source

Data for this brief communication were derived from a previously published systematic review with meta-analysis of prospective cohort studies that included an examination of the effects of LTPA on risk of stroke in adults, details of which have been described elsewhere [6]. Briefly, this previous systematic review with meta-analysis included nine prospective cohort studies that examined the association between LTPA and the relative risk (RR) of fatal or nonfatal stroke (hemorrhagic or ischemic) in an initial population of 269,594 men and women 25 to 84 years of age [6]. Follow-up periods ranged from 7.7 to 32 years (mean ± SD = 13.4 ± 7.8, Median = 11.1) [6]. Leisure time physical activity was assessed using a variety of methods and was categorized in the original meta-analysis as either low (reference), moderate, or high, with categories between the lowest and highest levels pooled to represent moderate LTPA [6]. This created two groups of comparisons, moderate versus low and high versus low, with data reported separately for men and women [6].

### 2.2. Data Synthesis

#### 2.2.1. Effect Size Calculations

The effect sizes pooled for the current study were extracted directly from previously reported RR and 95% confidence intervals examining the association between LTPA and stroke [6]. Effect sizes were reported according to sex (men and women) and type of comparison (moderate versus low PA and high versus low PA).

#### 2.2.2. Effect Size Pooling

The recently developed IVhet model [8] was used to pool RR results for the association between LTPA and risk of stroke. Details regarding the model are provided in the original article [8]. Briefly, the IVhet model is a quasi-likelihood model that is calculated by (1) calculating weights that sum to 1 for each study, (2) pooling effects from all the studies, and (3) calculating the variance of the pooled RR [8]. It has been shown to be more robust than the traditional random-effects model of Dersimonian and Laird [8]. For the current study, all analyses were conducted using the log transformation and then back transformed to RR for presentation purposes. A two-tailed alpha value ≤ 0.05 and nonoverlapping 95% confidence intervals were considered statistically significant. Results were then compared to previously reported results based on the random-effects model used in the original meta-analysis [8]. Heterogeneity and inconsistency, results of which were not reported in the original meta-analysis [8] were examined using the Q and* I*^2^ statistic, respectively, with an alpha value ≤ 0.10 for Q considered to represent statistically significant between-study heterogeneity. For* I*^2^, inconsistency was considered to be very low (<25%), low (25% to <50%), moderate (50% to <75%), or large (≥ 75%) [12]. Absolute between-study heterogeneity was reported using tau-squared (*τ*^2^). Small-study effects (publication bias, etc.) were examined according to the general guidelines of Sterne et al. [9]. In addition, a recently developed and robust approach was used to provide both a graphical (Doi plot) and quantitative [Luis Furuya-Kanamori (LFK) index] examination for potential small-study effects [10]. Luis Furuya-Kanamori indices of ± 1, between ± 1 and ± 2, and > ± 2 were considered to represent no, minor, and major asymmetry, respectively [13]. Given the small number of studies included, influence analysis was conducted with each study deleted from the model once in order to see if there was an effect on the overall results. In addition, cumulative meta-analysis, ranked by year, was conducted in order to examine the influence of findings over time. All analyses were performed using Meta XL, version 5.3 [13].

## 3. Results

### 3.1. Moderate versus Low Leisure Time Physical Activity

**Figure 1:**
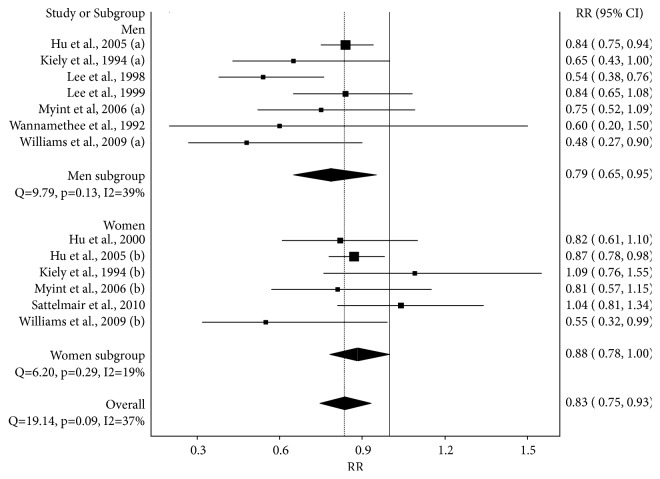
Forest plot for the association between moderate versus low leisure time physical activity and the relative risk of fatal or nonfatal stroke (hemorrhagic or ischemic) using the IVhet model. The black squares represent the relative risks (RR) while the left and right extremes of the squares represent the corresponding 95% confidence intervals for the RR. The middle of the black diamonds represents the RR while the right and left extremes of the diamond represent the corresponding 95% confidence intervals.

A forest plot of the association between moderate versus low LTPA is shown in Figure 1 and Supplementary File 1. Overall, moderate LTPA was associated with a statistically significant RR reduction of 17% in the risk for stroke (p = 0.002). Statistically significant heterogeneity was observed but inconsistency was considered low. Tau-squared was 0.01. Small-study effects were considered to be major (LFK index, -3.44, Supplementary File 2). With each study deleted from the model once, results remained statistically significant, ranging from a RR reduction of 18% (RR = 0.82, 95% CI = 0.71 to 0.95) to 15% (RR = 0.85, 95% CI 0.78 to 0.92) (Supplementary Files 3 and 4). Cumulative meta-analysis, ranked by year, revealed that results have remained statistically significant and stable since the year 2005 (Supplementary Files 5 and 6). When partitioned according to gender, a statistically significant RR reduction of 21% (p = 0.01) was observed in men while a trend for a statistically significant RR reduction of 12% (p = 0.05) was observed in women (Figure 1 and Supplementary File 1). Pooled 95% CI overlapped between the men and women subgroups (Figure 1 and Supplementary File 1). Compared to the previous meta-analysis using the less robust random-effects model [6], the RR reduction for men were 6% smaller (21% versus 27%) with wider 95% CI (30% versus 23%). For women, the RR reduction was approximately the same (12% versus 11%) with similar widths for the 95% CI (22% versus 21%).

### 3.2. High versus Low Leisure Time Physical Activity

**Figure 2:**
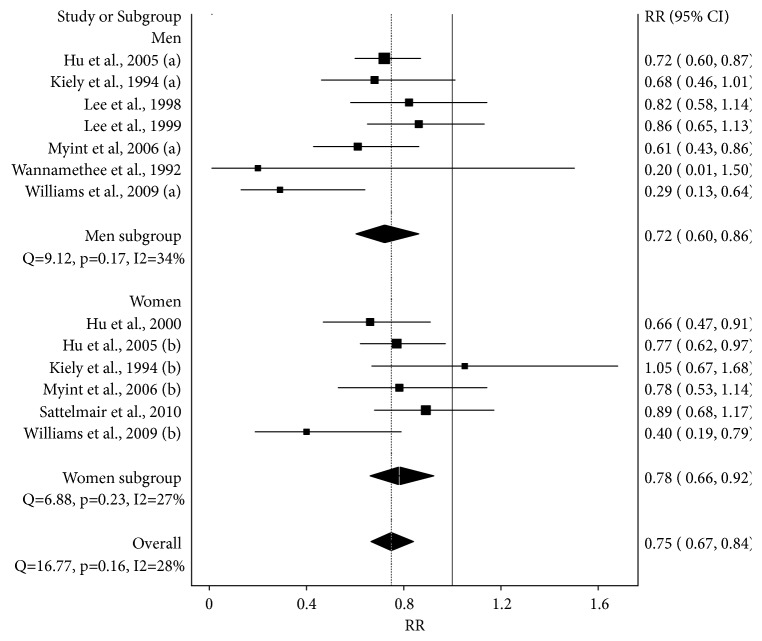
Forest plot for association between high versus low leisure time physical activity and the relative risk of fatal or nonfatal stroke (hemorrhagic or ischemic) using the IVhet model. The black squares represent the relative risks (RR) while the left and right extremes of the squares represent the corresponding 95% confidence intervals for the RR. The middle of the black diamonds represents the RR while the right and left extremes of the diamond represent the corresponding 95% confidence intervals.

A forest plot of the association between high versus low LTPA is shown in Figure 2 and Supplementary File 7. Overall, high LTPA was associated with a statistically significant RR reduction of 25% in the risk for stroke (p < 0.001). No statistically significant heterogeneity was observed and inconsistency was considered low. Tau-squared was 0.01. Small-study effects were considered to be major (LFK index, -3.00, Supplementary File 8).

With each study deleted from the model once, results remained statistically significant, ranging from a RR reduction of 27% (RR = 0.73, 95% CI = 0.65 to 0.83) to 24% (RR = 0.76, 95% CI 0.67 to 0.86) (Supplementary Files 9 and 10). Cumulative meta-analysis, ranked by year, revealed that results have remained statistically significant and stable since the year 2005 (Supplementary Files 11 and 12). When partitioned according to gender, a statistically RR reduction of 28% (p = 0.01) was observed in men while a statistically significant RR reduction of 22% (p = 0.05) was observed in women (Figure 2 and Supplementary File 7). Pooled 95% CI overlapped between the two subgroups (Figure 2 and Supplementary File 7). Compared to the previously published meta-analysis using the less robust random-effects model [6], the RR reduction for men was approximately the same for the overall RR (28% versus 29%) as well as the width of 95% CI (26% versus 24%). For women, the RR reduction was the same for both the overall RR (22%) and widths for the 95% CI (26%).

## 4. Discussion

### 4.1. Overall Findings

The overall results of the current meta-analysis suggest that both moderate and high LTPA reduce the RR of stroke in adults. These findings are supported by (1) the statistically significant effects observed in both men and women, (2) the lack of statistically significant heterogeneity and/or inconsistency, (3) the lack of influence of any one study on the overall results, and (4) the stability of findings since at least the year 2005. In contrast, the major asymmetry observed suggests the presence of small-study effects, possibly publication bias. This implies that the current findings may be an overestimate of the true effects, including the possible direction, of the association between moderate and high LTPA and incident stroke in men and women. While methods of adjusting for such asymmetry exist, none are highly recommended [9].

From the investigative team's perspective, the current study provides more robust information than the previous meta-analysis with respect to the association between LTPA and incident stroke [6]. More specifically, the association between the RR of stroke and moderate LTPA in men was approximately 6% less in the current meta-analysis. In addition, the current study identified small-study effects that were not observed in the previous meta-analysis [6]. Thus, while both studies suggest that moderate and high LTPA reduce the risk of incident stroke, the current findings are more conservative as well as robust.

### 4.2. Implications for Research

There are at least two implications for research. First, given the stability of results for approximately 13 years, there may not be a need from a public health perspective to continue conducting research on the effects of physical activity on incident stroke in adult men and women. However, there is a need for precision-medicine research to identify the appropriate dose of exercise for preventing stroke at the level of the individual. Second, a need exists for additional research on a valid, user-friendly approach for adjusting for small-study effects (publication bias, etc.), a major problem in published research [14], including stroke research [15].

### 4.3. Implications for Practice

Despite the inability to examine for the dose-response effects of physical activity at either the group or individual level, it would appear plausible to suggest that adherence to current physical activity guidelines would decrease the risk for stroke at the population level [16]. This includes 150 minutes per week of moderate intensity activity (brisk walking, etc.), 75 minutes per week of vigorous intensity aerobic activity (jogging, etc.), or a comparable combination of moderate and vigorous intensity aerobic activity [16].

### 4.4. Strengths and Potential Limitations

From our perspective, there are at least three strengths to the current study. First, a more robust model, the IVhet model [8], was used to arrive at more valid results regarding LTPA and the incidence of stroke in men and women. Second, a more robust approach was used to provide both a qualitative and quantitative examination for small-study effects [10]. Third, additional statistics not assessed and/or reported in the original meta-analysis [6] provided more robust information regarding LTPA and stroke in men and women. These included data on (1) heterogeneity and inconsistency, (2) the influence of each study on the overall results, and (3) the stability of findings over time.

While there are several strengths to the current study, there are at least four potential limitations. First, and as reported in the original meta-analysis [6], the inability to examine for ischemic and hemorrhagic stroke separately may have yielded different findings between the two. However, previous and earlier meta-analytic work on LTPA and stroke found no statistically significant differences between the two [17, 18]. Second, given that the data were based on self-report from different physical activity questionnaires, the potential for compromised results exist [19]. Third, the previous [6] and current meta-analysis was limited to LTPA and thus, did not account for occupational physical activity. Fourth, since the current findings were based on aggregate data, there is the potential for ecological fallacy [20].

## 5. Conclusions

The results of this study suggest that LTPA is associated with a reduced risk of stroke in both men and women. However, the small-study effects observed suggest the possibility that results may be exaggerated.

## Data Availability

The data used to support the findings of this study are available from the corresponding author upon request.

## Supplementary Materials

Supplementary Materials Supplementary File 1: Forest plot for the association between moderate versus low leisure time physical activity and the relative risk of fatal or nonfatal stroke (hemorrhagic or ischemic) using the IVhet model. The black squares represent the log scale relative risks (In RR) while the left and right extremes of the squares represent the corresponding 95% confidence intervals for the In RR. The middle of the black diamonds represents the In RR while the right and left extremes of the diamond represent the corresponding 95% confidence intervals. Supplementary File 2: Doi plot of overall results for moderate versus low leisure time physical activity. Supplementary File 3: Influence analysis plot of overall results for moderate versus low leisure time physical activity with each study deleted from the model once and ranked from largest to smallest reductions in the overall RR. Supplementary File 4: Influence analysis plot (log scale) of overall results for moderate versus low leisure time physical activity with each study deleted from the model once and ranked from largest to smallest reductions in the overall RR. Supplementary File 5: Cumulative meta-analysis, ranked by year, of overall RR results for moderate versus low leisure time physical activity. Supplementary File 6: Cumulative meta-analysis, ranked by year, of overall RR (log scale) results for moderate versus low leisure time physical activity. Supplementary File 7: Forest plot for the association between high versus low leisure time physical activity and the relative risk of fatal or nonfatal stroke (hemorrhagic or ischemic) using the IVhet model. The black squares represent the log scale relative risks (In RR) while the left and right extremes of the squares represent the corresponding 95% confidence intervals for the In RR. The middle of the black diamonds represents the In RR while the right and left extremes of the diamond represent the corresponding 95% confidence intervals. Supplementary File 8: Doi plot of overall results for high versus low leisure time physical activity. Supplementary File 9: Influence analysis of overall results for high versus low leisure time physical activity with each study deleted from the model once and ranked from largest to smallest reductions in the overall RR. Supplementary File 10: Influence analysis of overall results for high versus low leisure time physical activity with each study deleted from the model once and ranked from largest to smallest reductions in the overall RR on the log scale. Supplementary File 11: Cumulative meta-analysis, ranked by year, of overall RR results for high versus low leisure time physical activity. Supplementary File 12: Cumulative meta-analysis, ranked by year, of overall RR (log scale) results for high versus low leisure time physical activity.
