# Supplementary material for: Leisure Time Physical Activity Reduces the Risk for Stroke in Adults: A Reanalysis of a Meta-Analysis Using the Inverse-Heterogeneity Model
Source: Stroke Res Treat. 2019 Jun 2;2019:8264502. doi: 10.1155/2019/8264502 (PMC6582787; doi:10.1155/2019/8264502)

Supplementary Files

Supplementary file 1. Forest plot for the association between moderate versus low leisure time physical activity and the relative risk of fatal or non-fatal stroke (hemorrhagic or ischemic) using the IVhet model. The black squares represent the log scale relative risks (ln RR) while the left and right extremes of the squares represent the corresponding 95% confidence intervals for the ln RR. The middle of the black diamonds represent the ln RR while the right and left extremes of the diamond represent the corresponding 95% confidence intervals.

Supplementary file 2. Doi plot of overall results for moderate versus low leisure time physical activity.

Supplementary file 3. Influence analysis plot of overall results for moderate versus low leisure time physical activity with each study deleted from the model once and ranked from largest to smallest reductions in the overall RR.

Supplementary file 4. Influence analysis plot (log scale) of overall results for moderate versus low leisure time physical activity with each study deleted from the model once and ranked from largest to smallest reductions in the overall RR.

Supplementary file 5. Cumulative meta-analysis, ranked by year, of overall RR results for moderate versus low leisure time physical activity.

Supplementary file 6. Cumulative meta-analysis, ranked by year, of overall RR (log scale) results for moderate versus low leisure time physical activity.

Supplementary file 7. Forest plot for the association between high versus low leisure time physical activity and the relative risk of fatal or non-fatal stroke (hemorrhagic or ischemic) using the IVhet model. The black squares represent the log scale relative risks (ln RR) while the left and right extremes of the squares represent the corresponding 95% confidence intervals for the ln RR. The middle of the black diamonds represent the ln RR while the right and left extremes of the diamond represent the corresponding 95% confidence intervals.

Supplementary file 8. Doi plot of overall results for high versus low leisure time physical activity.

Supplementary file 9. Influence analysis of overall results for high versus low leisure time physical activity with each study deleted from the model and ranked from largest to smallest reductions in the overall RR.

Supplementary file 10. Influence analysis of overall results for high versus low leisure time physical activity with each study deleted from the model once and ranked from largest to smallest reductions in the overall RR on the log scale.

Supplementary file 11. Cumulative meta-analysis, ranked by year, of overall RR results for high versus low leisure time physical activity.

Supplementary file 12. Cumulative meta-analysis, ranked by year, of overall RR (log scale) results for high versus low leisure time physical activity.

Supplementary file 1

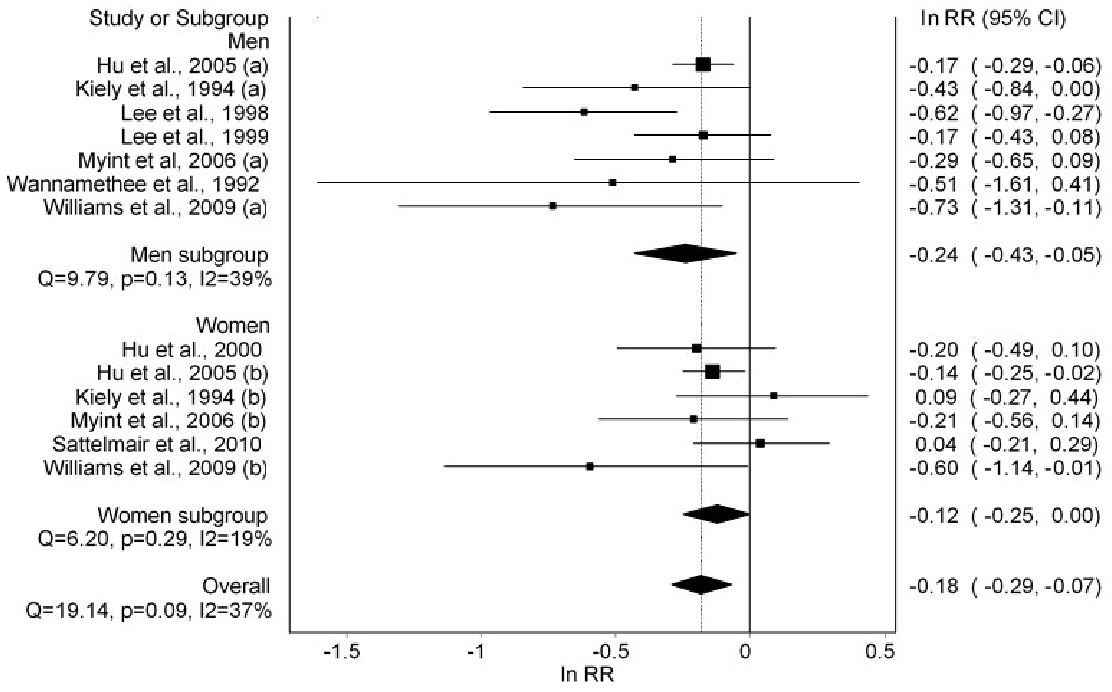

Supplementary file 2

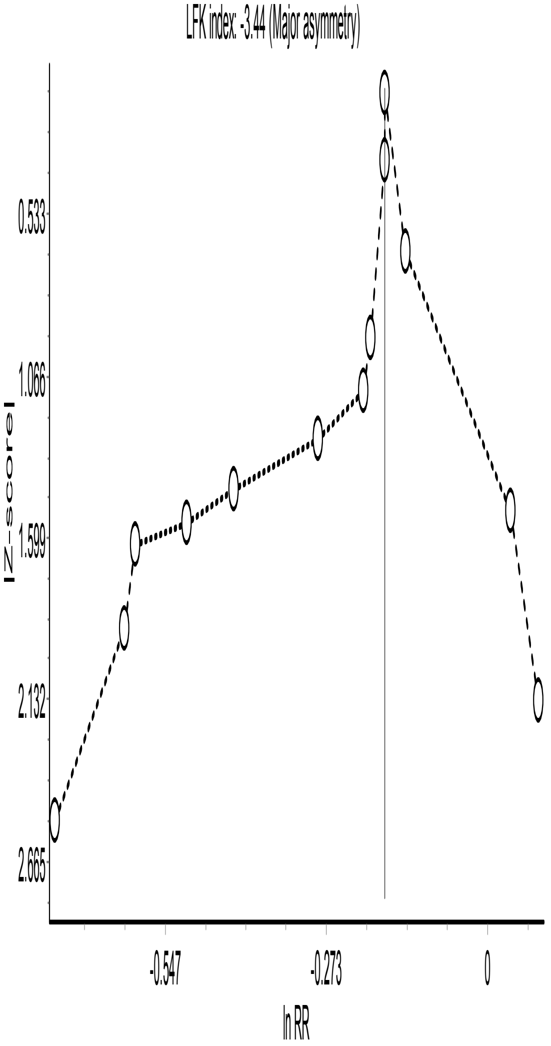

Supplementary file 3

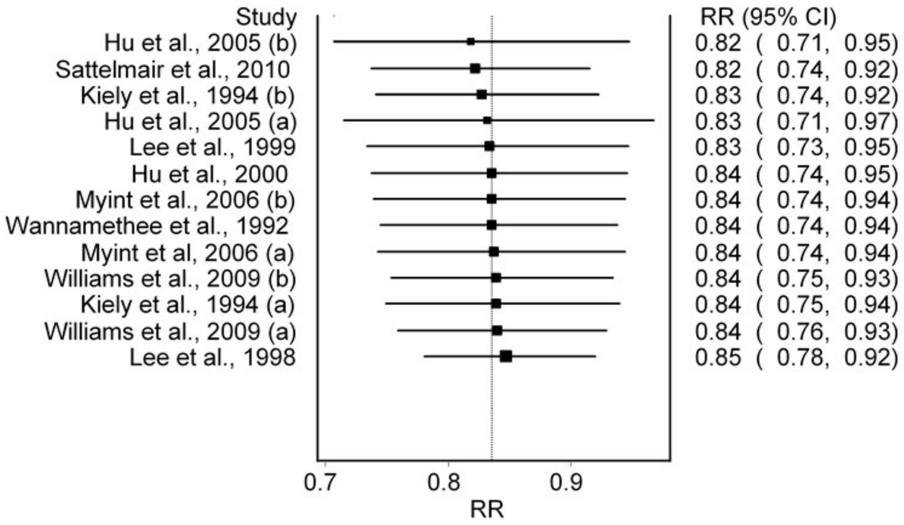

Supplementary file 4

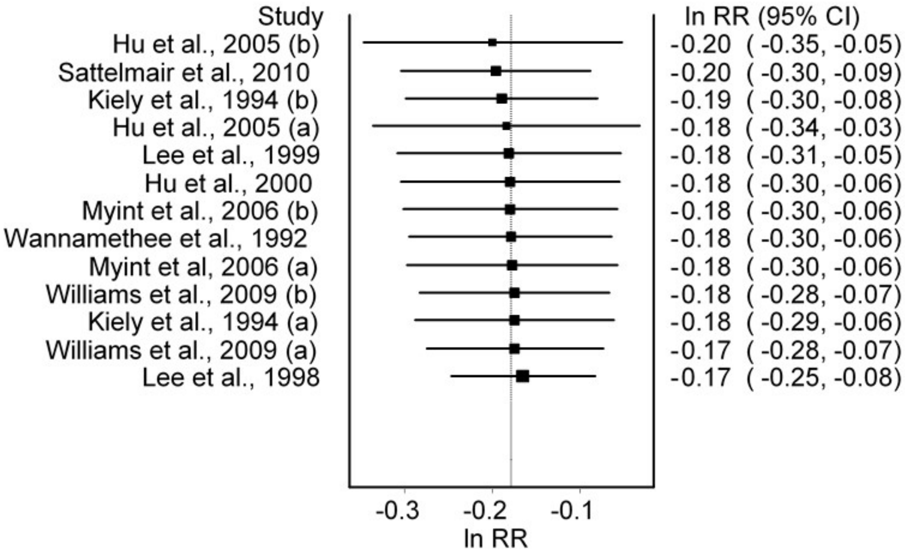

Supplementary file 5

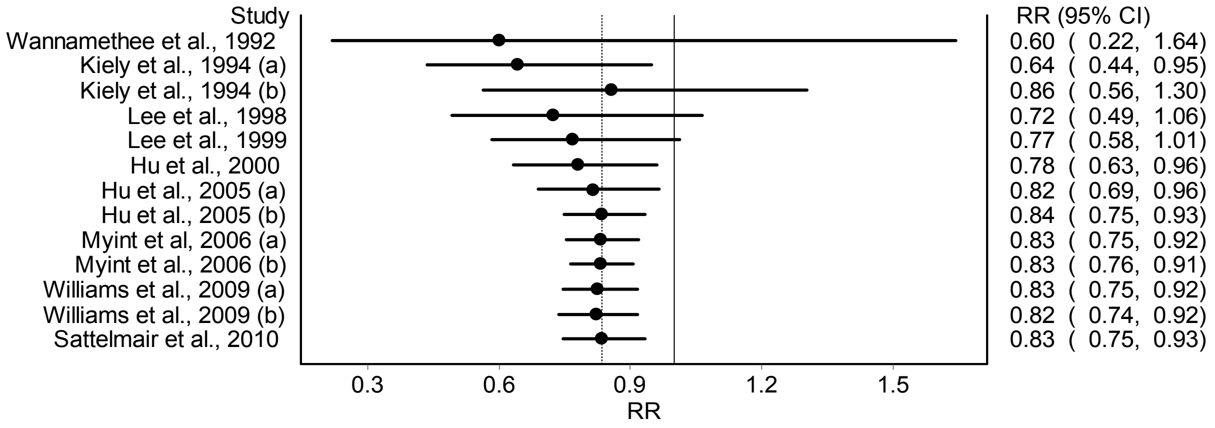

Supplementary file 6

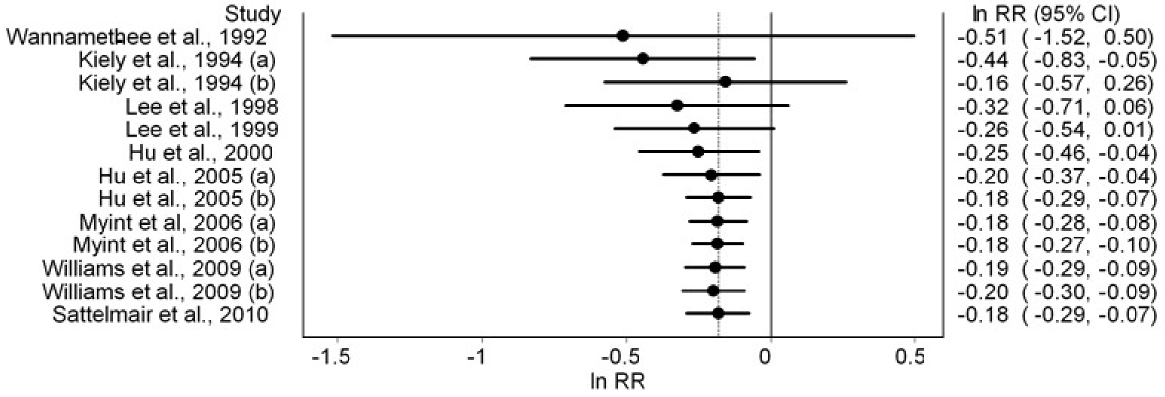

Supplementary file 7

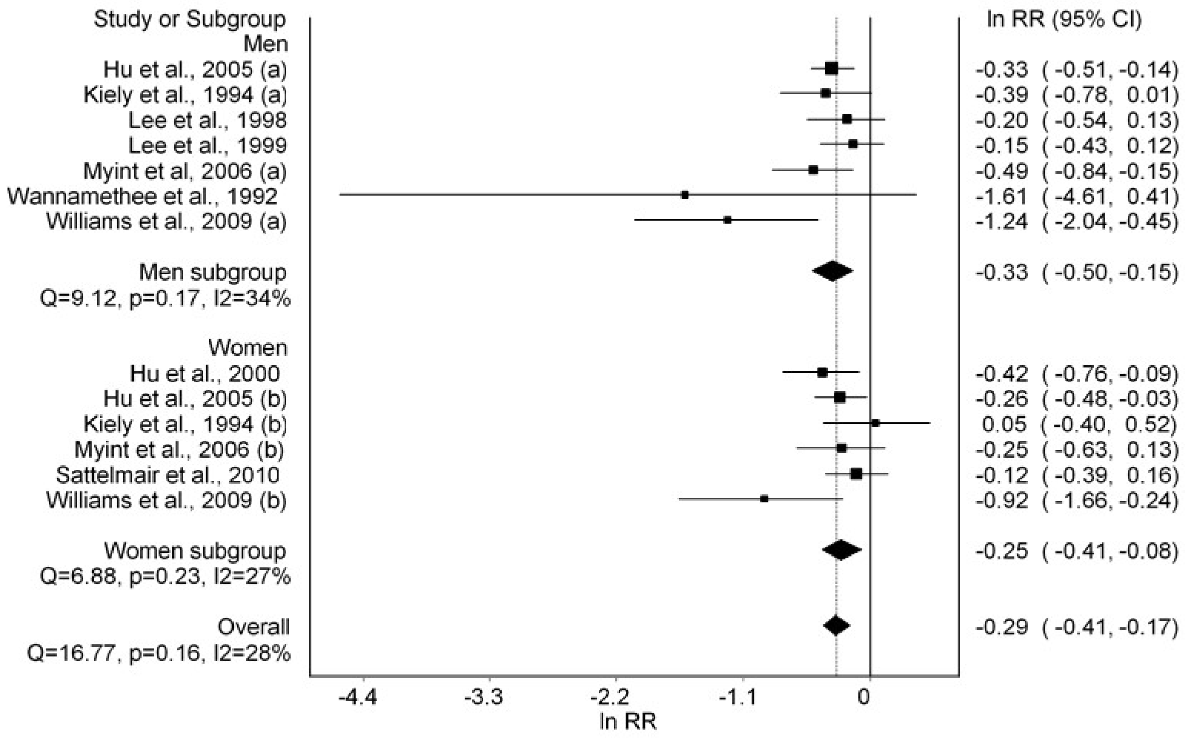

Supplementary file 8

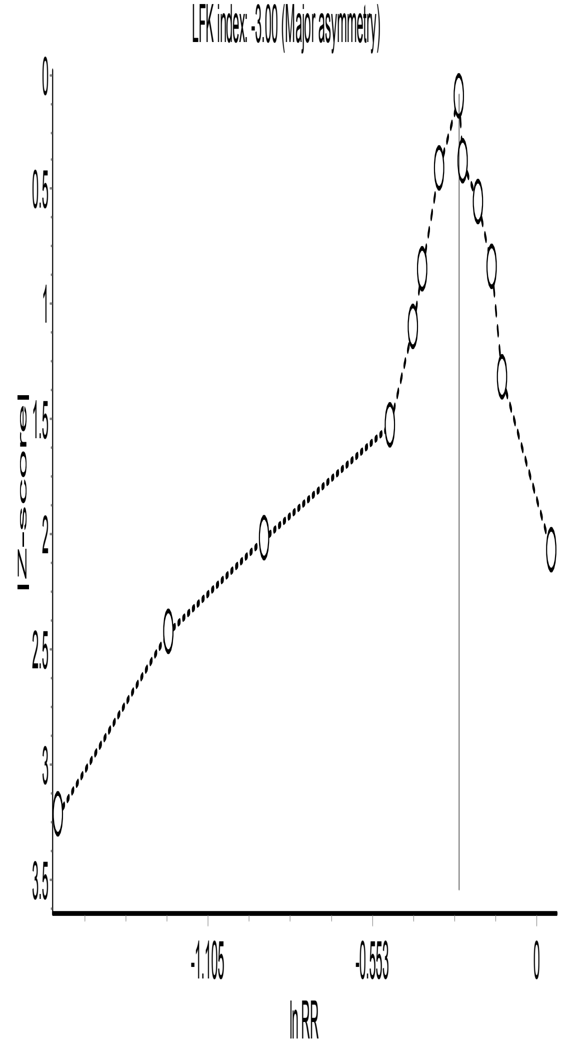

Supplementary file 9

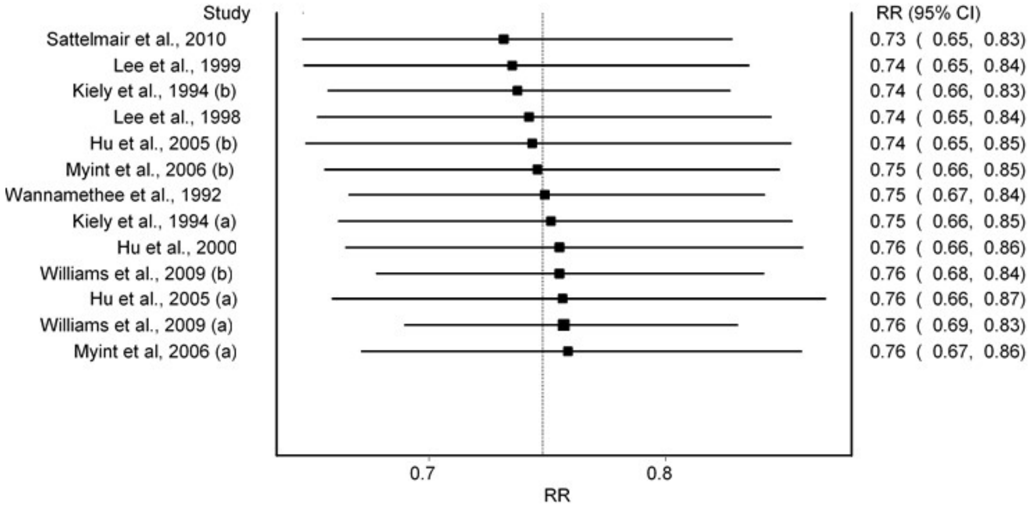

Supplementary file 10

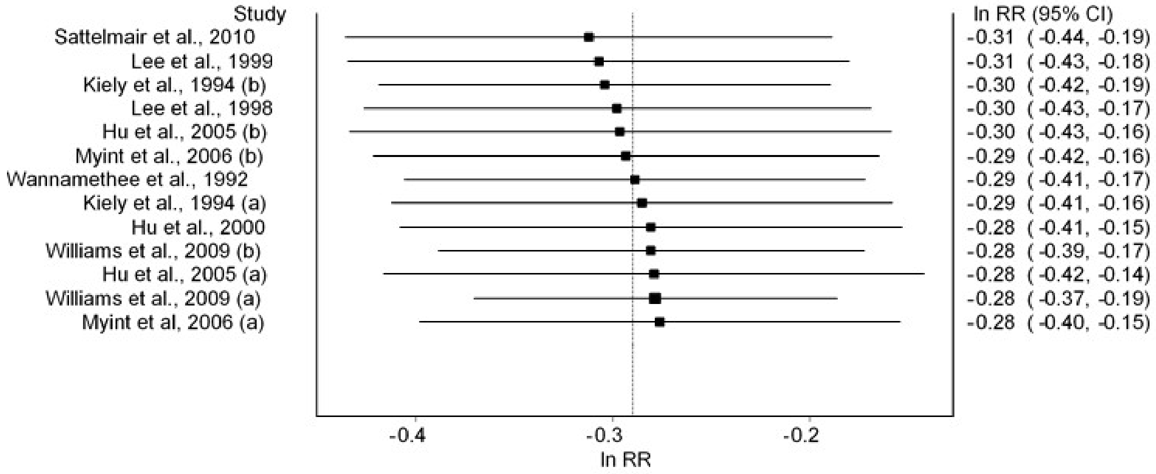

Supplementary file 11

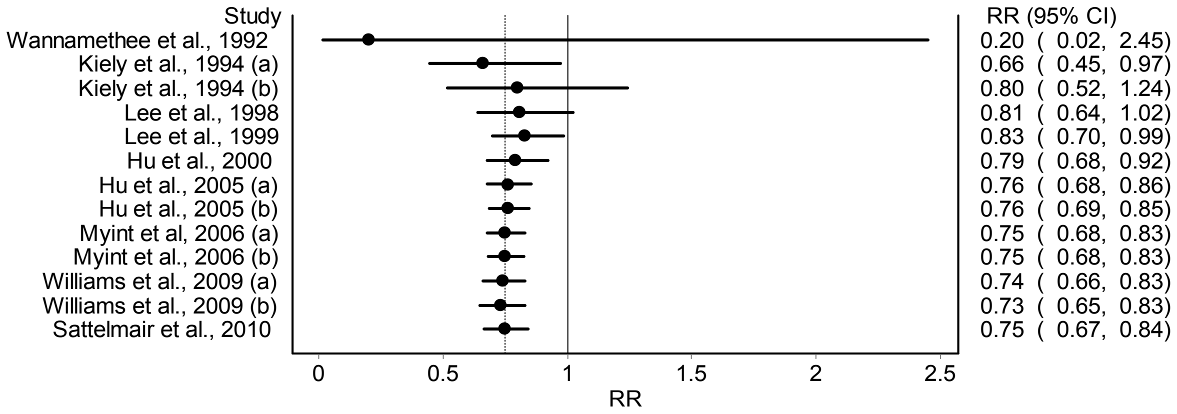

Supplementary file 12

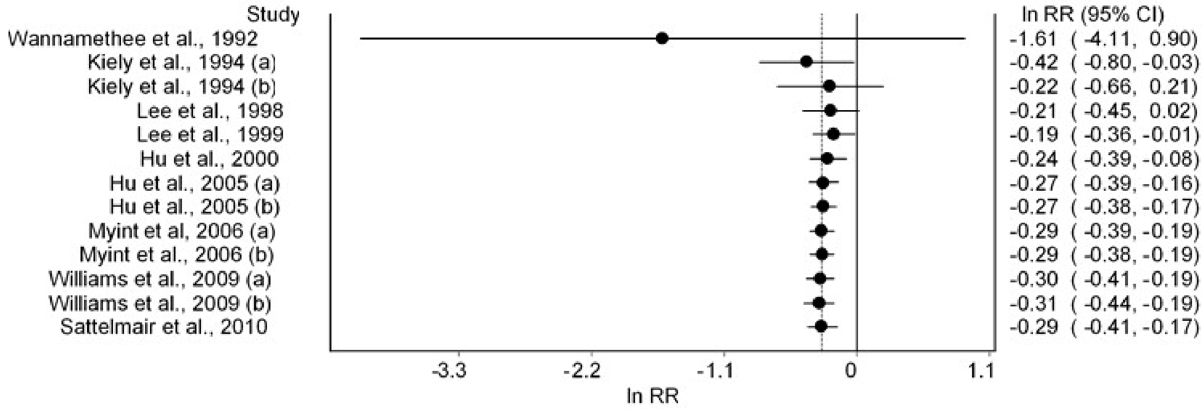

Supplement: Supplementary Materials — Supplementary File 1: Forest plot for the association between moderate versus low leisure time physical activity and the relative risk of fatal or nonfatal stroke (hemorrhagic or ischemic) using the IVhet model. The black squares represent the log scale relative risks (In RR) while the left and right extremes of the squares represent the corresponding 95% confidence intervals for the In RR. The middle of the black diamonds represents the In RR while the right and left extremes of the diamond represent the corresponding 95% confidence intervals. Supplementary File 2: Doi plot of overall results for moderate versus low leisure time physical activity. Supplementary File 3: Influence analysis plot of overall results for moderate versus low leisure time physical activity with each study deleted from the model once and ranked from largest to smallest reductions in the overall RR. Supplementary File 4: Influence analysis plot (log scale) of overall results for moderate versus low leisure time physical activity with each study deleted from the model once and ranked from largest to smallest reductions in the overall RR. Supplementary File 5: Cumulative meta-analysis, ranked by year, of overall RR results for moderate versus low leisure time physical activity. Supplementary File 6: Cumulative meta-analysis, ranked by year, of overall RR (log scale) results for moderate versus low leisure time physical activity. Supplementary File 7: Forest plot for the association between high versus low leisure time physical activity and the relative risk of fatal or nonfatal stroke (hemorrhagic or ischemic) using the IVhet model. The black squares represent the log scale relative risks (In RR) while the left and right extremes of the squares represent the corresponding 95% confidence intervals for the In RR. The middle of the black diamonds represents the In RR while the right and left extremes of the diamond represent the corresponding 95% confidence intervals. Supplementary File 8: Doi pl [file 8264502.f1.pdf]
